# Effect of artificial barriers on the distribution of the invasive signal crayfish and Chinese mitten crab

**DOI:** 10.1038/s41598-019-43570-3

**Published:** 2019-05-10

**Authors:** Chloe Victoria Robinson, Carlos Garcia de Leaniz, Sofia Consuegra

**Affiliations:** Bioscience Department, Swansea University, Singleton Park, Swansea, United Kingdom

**Keywords:** Invasive species, Molecular ecology

## Abstract

The role of river obstacles in preventing or facilitating the dispersal and establishment of aquatic invasive species is controversial. Novel detection tools like environmental DNA (eDNA) can be used for monitoring aquatic invasive species (AIS) such as the American signal crayfish (*Pacifastacus leniusculus*) and the Chinese mitten crab (*Eriocheir sinensis*), providing information on the effect of barriers on their distribution. We analysed eDNA from both water and surface sediment in three river catchments (Medway, Dee and Stour; Great Britain), with differing levels of connectivity, to determine spatial distribution of the two species, and assessed the effect of barriers on their eDNA detection. Positive eDNA detections were obtained within confirmed sites for both species in all catchments, with evidence of species overlap in the River Medway. Upstream barriers in the Medway positively influenced detection success of mitten crab lower in the catchment while detection success of signal crayfish was higher in the highly fragmented catchment (River Medway). This information on the role of river barriers on AIS distribution and eDNA detection is important for management strategies and for predicting both future dispersal and likelihood of new colonisations in previously uninvaded fragmented catchments.

## Introduction

The introduction of aquatic invasive species (AIS) within the last century has been largely influenced by the expansion of aquaculture^1,2^ and the lack of adequate ballast water treatment^3,4^. The successful dispersal and establishment of AIS often results in negative consequences for native biota, through competition for resources, introduction of novel pathogens and through habitat transformation and/or degradation^5^. Thus, understanding the factors affecting the spread of invasive species is critical, and the influence of anthropogenic activities and man-made structures needs to be considered in addition to the ecological capabilities of the species^6–8^.

Fragmentation of aquatic systems as a consequence of the presence of man-made structures such as roads, locks and culverts, or through natural barriers such as waterfalls has direct impacts on both native and non-native biota^9^. Habitat alteration and associated stressors acting on native species in fragmented ecosystems are likely to facilitate the establishment of AIS compared to fully connected habitats^10^. The installation of barriers can cause fundamental changes in the lotic ecosystems, including reduction in flow variability^11^ and fine sediment accumulation upstream of the barrier^12^, which often can remove native species at a local scale through either stress or dispersal to a more favourable environment, thus opening a niche for invading species^13^. Dams create novel impoundments, where AIS can be up to 300 times more likely to occur than in natural lakes^6^. Additionally, impoundments are considered to act as a ‘bridge’ habitat in some cases, increasing the risk of invasion of natural lentic systems by residing AIS within close proximity^6^. River barriers may act as an efficient barrier for solely aquatic invaders, however the influence on AIS which are not limited to movement through water is rarely considered^14,15^.

The North American signal crayfish (*Pacifastacus leniusculus*) and the Chinese mitten crab (*Eriocheir sinensis*) represent two of the most successful AIS in the world but the factors determining their dispersal success are largely unknown. Human-mediated dispersal has contributed to the expansion of the invasive signal crayfish, which was intentionally imported and farmed in Great Britain from 1970 to 1990^16^. Signal crayfish is a voracious invader, introduced through a combination of purposeful stocking implants and escape events^17^ and has caused a 90% decline in native white-clawed crayfish (*Austropotamobius pallipes*) through competition and transmission of crayfish plague (*Aphanomyces astaci*)^6,18^. The Chinese mitten crab, which is becoming increasingly abundant across Great Britain^19,20^ is an additional host of the crayfish plague pathogen. Ballast water and mariculture are the main vectors of introduction for mitten crabs and, similar to signal crayfish, mitten crabs are notorious for their destructive nature towards native biodiversity and banks and levees of lakes and rivers^21–23^.

Overland dispersal in invasive crayfish species has been reported numerous times^15,24–27^ in a number of species (e.g. red swamp crayfish (*Procambarus clarkii*; signal crayfish), including mitten crabs^28^. If faced with unfavourable conditions and/or barriers, crayfish and mitten crabs are known to exit the water to find more suitable habitats and to overcome barriers^15,24,27^. Although man-made cross-channel barriers and natural barriers (rapids and waterfalls) could in some cases restrict the upstream dispersal of crayfish^15,26,29,30^, signal crayfish have been reported to disperse downstream more often than upstream to colonise new locations^31,32^, and therefore it is unclear whether river barriers inhibit the natural movement and dispersal of this species^31,33^. In contrast, barriers such as dams are likely to impede the migration of mitten crabs, which tends to occur upstream, limiting the dispersal of the species^34^.

In the field, it is difficult to assess the relative effects of barriers on presence and dispersal of mitten crabs using traditional surveys such as direct observation and trapping^35,36^. Conventional traps are size-biased towards smaller individuals, often failing to trap mitten crabs with carapaces >19 mm^35,37^. Fyke nets have proven to be effective at catching mitten crabs when they are in large numbers^37^, however they pose problems with by-catch of native fish and mammals^37^. Thus, the inefficiency of conventional methods can result in false negatives when assessing the upstream migration of mitten crabs in relation to barriers. Trapping also has variable efficiencies in detecting signal crayfish^38^, but the development of novel molecular techniques (environmental DNA) has enabled fine-scale detection across a variety of waterbodies^39–43^.

The environmental DNA (eDNA) approach has been increasingly used for detection and, potentially, quantification of AIS, with eDNA successfully detected both in aqueous samples and in aquatic sediment and some studies suggest that DNA concentration is higher in sediments than in surface water^42,44–^^49^. In addition, eDNA has the potential to aid understanding of how river barriers can limit the upstream progression of a range of aquatic species, including invasive species^33^, and has been used to identify the successful upstream migration of migratory fishes over barriers^31,50^. One of the limitations of eDNA sampling in flowing systems is that the source of the extracellular DNA cannot be easily determined^51,52^. Invertebrate eDNA has previously been successfully detected up to 12 km downstream from the DNA source^53^ but the persistence of eDNA in riverine systems from source to sample site depends on numerous factors, including flow rate^51,53,54^. During periods of low flow, DNA is more likely to sink into the substratum and bind to the sediment, reducing the distance travelled downstream^55,56^ and potentially increasing the longevity of the DNA^47–49^.

Using eDNA methods, multiple species can be detected at once, either by using universal primers^57^ or by undertaking the multiplex approach^39,58^, by which rivers can be surveyed for presence/absence of target species simultaneously at various locations of the catchment. High resolution melt (HRM) profiling combined with eDNA quantitative PCR (qPCR) is an emerging analysis technique which allows the detection of single-base variations in DNA sequences by differences in double stranded DNA product melt temperature^39,59^. The PCR product melt temperature (tm) depends on the sequence composition, fragment length and the choice of qPCR MasterMix used in the PCR reaction^39,59^. The main advantage of using HRM analysis over conventional probe-based qPCR assays for eDNA presence/absence, is the ease of distinguishing non-target amplifications from true melt peaks of target species, which limits the rate of type I errors^60,61^. In addition, adopting the HRM approach allows the use of multiple primer pairs within the same qPCR reaction to detect more than one species simultaneously. This multiplexing approach has been previously implemented to detect a combination of signal crayfish, white-clawed crayfish and crayfish plague oomycete *A. astaci*, from eDNA samples^39^.

In this study, we assessed the presence of both signal crayfish and mitten crab within three catchments in Great Britain with different degrees of fragmentation, using different eDNA sample types (water and sediment). We aimed to investigate the potential of eDNA in identifying the effects of barrier presence on limiting the dispersal of these species, upstream in the case of mitten crabs (through determining the upstream limit of eDNA detection) and downstream in the case of the crayfish.

## Materials and Methods

### Sample sites and eDNA collection

**Figure 1:**
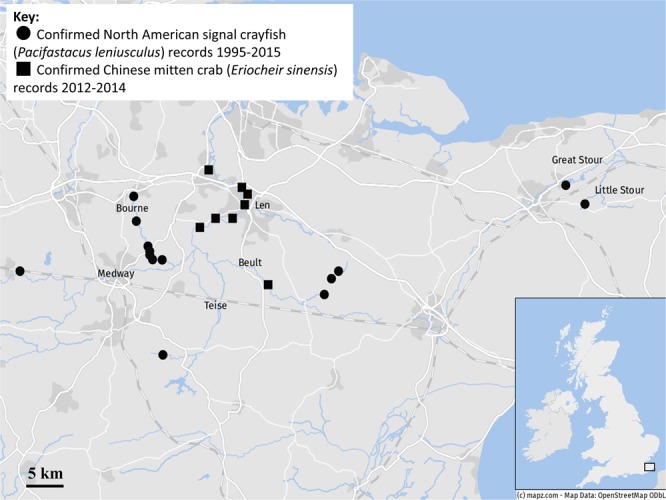
Distribution map for both North American signal crayfish (*Pacifastacus leniusculus*) and Chinese mitten crab (*Eriocheir sinensis*) in the River Medway and River Stour catchments, from 1995–2015 (signal crayfish) and 2012–2014 (mitten crabs). Data: ©Environment Agency, Map: © mapz.com.

**Figure 2:**
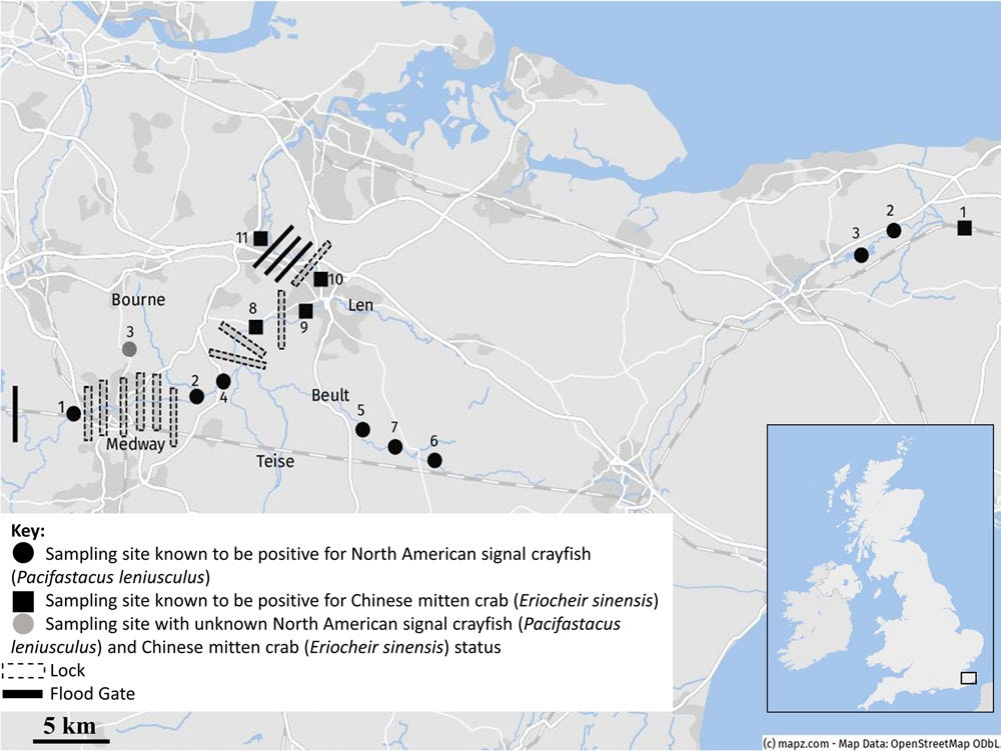
eDNA sampling sites for the Rivers Medway and Stour. Signal crayfish DNA was confirmed at sites 1 (Tonbridge Castle), 5 (Horse Farm), 7 (Green Lane), 8 (Teston Bridge), 10 (Asda), and 11 (Leybourne Lakes); mitten crab DNA was confirmed at sites 7, 10 and 11 in the Medway and in the Stour, both signal crayfish and mitten crab were detected at sites 7, 10 and 11. At each point, three water samples and between zero and three sediment samples were collected in 2016. © mapz.com.

**Figure 3:**
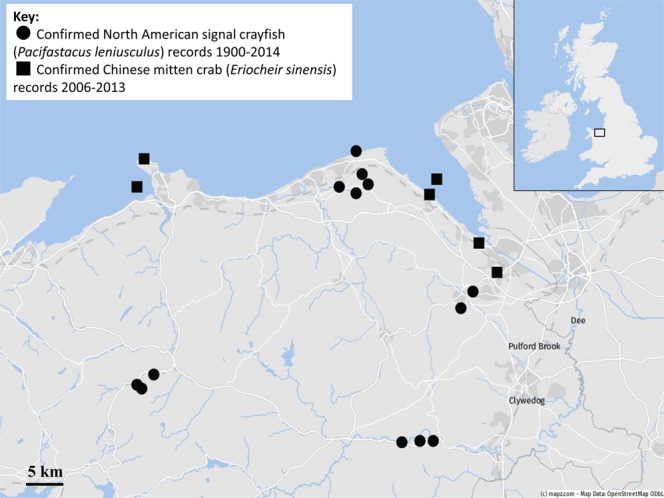
Distribution map for both North American signal crayfish (*Pacifastacus leniusculus*) and Chinese mitten crab (*Eriocheir sinensis*) in the River Dee catchments, from 1990–2014 (signal crayfish) and 2006–2013 (mitten crab). Data: ©NBN Atlas, Map: © mapz.com.

**Figure 4:**
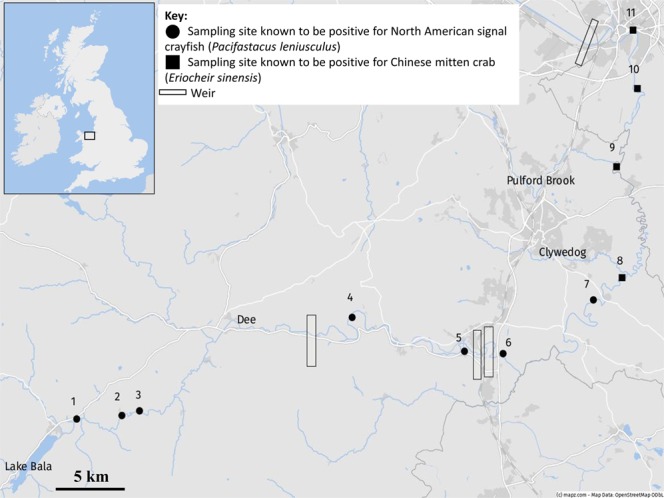
eDNA sampling sites for River Dee. Signal crayfish DNA was confirmed at sites 1 (NRW Bala), 7 (Sutton Green) and 8 (Caldecott); mitten crab DNA was confirmed at sites 7, 8 and 9 (Holt). At each point, six water samples and three sediment samples were collected in 2016. © mapz.com.

Samples were collected in July/October 2016 from three river systems: the River Medway (14 barriers; Figs 1 and 2; Table S1), the River Dee (four barriers; Figs 3 and 4; Table S1) and the River Stour (no barriers; Figs 1 and 2), with the assistance of North Wales Wildlife Trust (Dee). The River Medway spans 113 km from West Sussex to Sheerness^62^, the River Dee is considerably more connected than the Medway and runs 110 km from source at Dduallt to the Dee estuary^63^, and the River Stour is an unfragmented river that begins at Lenham and runs 82.4 km to Pegwell Bay^64^. Sampling took place in the high-mid to lower catchment of each river (Figs 2 and 4). These three river systems sustain populations of both signal crayfish and mitten crab, the former generally occupying the upper to middle reaches and the latter occupying the middle (Figs 1 and 3)^20,65^. These particular rivers were chosen due to their differing levels of fragmentation and because are only affected by artificial barriers.

A total of 11 sites were sampled in the River Medway and River Dee and three sites were sampled in the River Stour (Table 1). Samples were collected at regular intervals along all three rivers where possible, starting in the most downstream site so as not to bias results from disturbing sediment. Distance from each sampling site to nearest barrier upstream and downstream was measured for the Medway and Dee (Table S2), however as there are no known barriers in the Stour within the river segment sampled, distance was no calculated for this river. Three water samples of 30 mL were taken at each site (one on either side of river near bank and one in central channel), at a minimum of 1 m depth (where possible) for all river systems. After collection, 30 mL samples were split into two 15 mL samples, resulting in six samples per site. Each 15 mL sample was added to 33 mL of absolute ethanol and 1.5 mL 5 M sodium acetate in a 50 mL Falcon tube and tubes were subsequently placed on ice before being stored upright at −20 °C until DNA extraction. This method was based on previous eDNA work^66,67^, including our own studies using 15 mL water volume for detecting signal crayfish^39^, as well as other work on crayfish^42,59^ and several aquatic species^42,66,68^. Negative controls consisting of ultrapure water in place of DNA were taken both before sampling and at the end of each sampling effort per site to test for any DNA carryover between sites potentially resulting in false positives. In addition to water samples, two 5 g sediment samples were collected at each site where possible for all river systems. Due to lack of sediment cohesion at a majority of sites, a sterile 15 mL Falcon tube was used to collect 5 mL from the top 2 cm of sediment^49^. We collected eDNA water samples prior to collecting sediment samples, to ensure DNA being collected was derived from the water and not from re-suspension of fragments from the sediment during collection^49^. Sediment was stored on ice and then kept frozen at −80 °C until DNA extraction. Environmental conditions including temperature, flow rate, shade cover, bank consistency (concrete vs. mud/clay) and also bank angle (rounded up to nearest 5°) in relation to river/pond water were recorded for each site (Table 1).

**Table 1:** Site information for eDNA sample collection in the River Medway (M), River Stour (S) and River Dee (D) including site name, GPS coordinates of site, water temperature (°C), flow rate (m/s), shade level (0–3), sediment collection status (yes/no), bank consistency (concrete vs. mud/clay) and bank angle in relation to waterbody (left and right in relation to downstream direction of water flow).

Site	Site Name	Site Type	River System	GPS	Temp. (°C)	Flow Rate (m/s)	Shade (0–3)	Sediment collected? (yes/no)	Bank consistency	Bank angle left (°)	Bank angle right (°)
M1	Tonbridge Castle	River	Medway	TQ 59089 46489	17	0.2	0	No	Concrete	90	90
M2	Tudeley Brook	Stream	Medway	TQ 67472 48254	15	0.3	0	No	Mud/clay	75	55
M3	Puttenden Lake	Pond	Medway	TQ 60810 51347	17	N/A	2	Yes	Mud/clay	30	N/A
M4	Canoe Landing	River	Medway	TQ 68987 49924	18	0.2	1	No	Concrete	90	90
M5	Horse Farm	River	Medway	TQ 72866 48687	16	0.25	1	No	Mud/clay	70	80
M6	Summerhill Road	Stream	Medway	TQ 77297 46511	14	0.1	3	Yes	Mud/clay	85	85
M7	Green Lane	Stream	Medway	TQ 72843 45680	13	0.2	1	Yes	Mud/clay	65	70
M8	Teston Bridge	River	Medway	TQ 70880 53290	15	0.2	1	Yes	Mud/clay	90	90
M9	Farleigh Station	River	Medway	TQ 73478 53564	16	0.4	0	No	Concrete	90	90
M10	Asda	River	Medway	TQ 75665 55630	17	0.3	0	No	Concrete	90	90
M11	Leybourne Lakes	Lake	Medway	TQ 70192 59812	19	N/A	2	Yes	Mud/clay	30	N/A
ST1	Gore Street	Stream	Stour	TR 26937 63415	17	0.25	2	No	Mud/clay	55	50
ST2	Grove Ferry Road	River	Stour	TR 23499 63189	18	0.1	1	No	Mud/clay	65	70
ST3	Fordwich	River	Stour	TR 17922 59782	17	0.5	1	Yes	Concrete	90	90
D1	NRW Bala	River	Dee	SH 93341 35505	15	N/A	0	Yes	Mud/clay	25	55
D2	Cilan	River	Dee	SJ 02021 37388	15	N/A	1	Yes	Mud/clay	50	45
D3	Carrog	River	Dee	SJ 02080 37443	16	N/A	0	Yes	Mud/clay	20	60
D4	Llangollen Serpents	River	Dee	SJ 20486 43565	15	N/A	3	Yes	Mud/clay	85	50
D5	Halton Woods	River	Dee	SJ 29494 40857	14	N/A	2	Yes	Mud/clay	50	45
D6	Eyton Hall	River	Dee	SJ 36286 44256	13	N/A	1	Yes	Mud/clay	55	60
D7	Sutton Green	River	Dee	SJ 41383 47928	13	N/A	0	Yes	Mud/clay	50	25
D8	Caldecott	River	Dee	SJ 42500 51100	13	N/A	3	Yes	Mud/clay	55	70
D9	Holt	River	Dee	SJ 40307 56900	14	N/A	3	Yes	Mud/clay	60	65
D10	Eccleston	River	Dee	SJ 41592 62289	13	N/A	2	Yes	Mud/clay	70	65
D11	Chester Meadows	River	Dee	SJ 41701 66398	14	N/A	0	Yes	Mud/clay	65	90

### DNA extraction and qPCR optimisation

Previously designed primers for crayfish^39^ (ApalPlen16SF: 5′-AGTTACTTTAGGGATAACAGCGT-3′ and ApalPlen16SR: 5′-CTTTTAATTCAACATCGAGGTCG-3′) were used to amplify a 83 bp product of both target species. Primers were assessed *in vitro* for mitten crab using positive control tissue (leg muscle) from eight mitten crab individuals from three populations (Maidstone, Kent; Chester, Cheshire; Leeds, Yorkshire). Mitten crab DNA was extracted using Qiagen® DNeasy Blood and Tissue Kit (Qiagen, UK), eluted in 200 µl, and amplified in end-point PCR with the above primers using the following protocol: 95 °C for 3 min, followed by 40 cycles of 95 °C for 30 s, 61 °C for 30 s and 72 °C for 45 s with a final elongation step of 72 °C for 10 min. All amplified PCR products were checked for the correct amplicon sizes using a 2% agarose gel electrophoresis. To confirm the species identity, PCR products were analysed using Sanger Sequencing on an ABI Prism 277 DNA sequencer. Resulting sequences were aligned using BioEdit v. 5.0.9 (using the ClustalW program) and inputted to BLAST^69^.

### Mitten crab qPCR-HRM optimisation

Optimisation of the primers above has previously been undertaken for signal crayfish^39^. Here, specific *in vitro* testing of RT-qPCR-HRM analysis was performed for mitten crab DNA only using SsoFast EvaGreen^®^ qPCR Supermix (BioRad, UK). The cycling protocol was carried out using a Bio-Rad CFX96 Touch Real-Time PCR Detection System (Bio-Rad, UK) and began with 15 min of denaturation at 98 °C, followed by 40 cycles of 95 °C for 15 s and 61.5 °C for 30 s. After the 40 cycles, a HRM step was applied to the RT-qPCR reactions, which consisted of applying a temperature gradient ranging from 65 °C to 95 °C in 0.1 °C/10 s increments, to melt the amplified qPCR product for assessment of consistency of amplicon tm. Resulting efficiency value for mitten crab DNA at pre-determined annealing temperature (61.5 °C) was 105.8%, R^2^ = 0.997 (previously determined efficiency of 100.2%, R^2^ = 0.986 for signal crayfish, and 107.9%^39^). Limit of detection (LOD) and limit of quantification (LOQ) were determined for mitten crab by running a dilution series ranging from 5 ng/µl to 5 × 10^−7^ ng/µl, using a mitten crab DNA pool. HRM analysis for mitten crab DNA was conducted on seven individuals to account for any degree of intraspecific variation in qPCR product tm. Overall, melt curves generated from species-specific product tms (signal crayfish: 73.8 °C ± 0.2; mitten crab: 73.2 °C ± 0.2) were analysed to assess the presence/absence of all species.

For assessing the ability to detect both target invasive species in the same reaction, different volume ratios were combined for the two target species (signal crayfish and mitten crab from 1:9 µl through to 9:1 µl signal crayfish: mitten crab DNA at 5 ng/µl) and amplified in triplicate.

### Analysis of eDNA field samples

DNA extraction was performed using Qiagen® DNeasy Powerlyzer PowerSoil Kit (Qiagen, UK), for both field eDNA water samples (n = 177; Table 2) and sediment eDNA samples (n = 39; Table 2), including negative controls, following the manufacturer’s instructions, apart from a reduction in the elution volume from 60 µl to 50 µl, to maximise DNA yield. We opted for Qiagen® DNeasy Powerlyzer PowerSoil Kit for all samples based on the effectiveness of the kit to remove inhibitors and produce high DNA yields^70,71^. Sediment samples were extracted in triplicate, resulting in a total of 117 sediment extractions. DNA extractions were undertaken in a dedicated eDNA area within an extraction cabinet, fully equipped with flow-through air system and UV light and to minimise contamination; additionally, dedicated eDNA laboratory coat and nitrile gloves were worn during the process.

**Table 2:** Catchment location, number of known river obstructions within area sampled, month/year of sample collection, number of sites sampled in 2016, total number of eDNA water and eDNA sediment samples collected from the Rivers Medway, Dee and Stour.

River System	Location	Number of Known River Obstructions	Month/Year	Number of sites sampled	Total number of eDNA water samples collected*	Total number of eDNA sediment samples collected
Medway	SE England	15	July/2016	11	78	18
Dee	N Wales	4	September/2016	11	78	18
Stour	SE England	0	July/2016	3	21	3
			TOTAL	**25**	**177**	**39**

*Including field blanks.

Amplifications were undertaken in triplicate using the protocol previously described, with the final optimised qPCR reactions carried out in a final volume of 10 µl, containing 2 µl of SsoFast™ EvaGreen® (Bio-Rad, UK), 0.25 µl of each primer (10 µM), 1 µl of template DNA at 5 ng/µl and 3.5 µl of ultrapure water. Melt curves generated from species-specific product tms (signal crayfish: 73.8 °C ± 0.2; mitten crab: 73.2 °C ± 0.2) were analysed to assess the presence/absence of target species in field samples. Samples which had at least two out of three PCR replicates with corresponding target tm for either or both species, with a melt rate above 200 -d(RFU)/dT were considered positive. In addition, qPCR reactions were carried out at sites positive for either signal crayfish or mitten crab (or both at same site) to test for presence of crayfish plague causal agent *A*. *astaci* using AphAstITS primers (Vrålstad *et al*. 2009). Each reaction consisted of 2 µl of 5 × HOT FIREPol^®^ EvaGreen^®^ qPCR Mix Plus ROX (Soils Biodyne, Estonia), 0.4 µl of primer mix (5 µM), 1 µl of template DNA at 5 ng/µl and 6.6 µl of ultrapure water^39^. Resulting melt peaks for target species using the HOT FIREPol^®^ EvaGreen^®^ qPCR Mix were 75.9 ± 0.2 °C (signal crayfish), 75.3 ± 0.2 °C (mitten crab) and 82.9 °C (*A*. *astaci*) respectively. qPCR amplifications were carried out in a separate room to eDNA extractions under a PCR hood with laminar flow. Each plate had the addition of both target species positive control DNA once all the eDNA samples were loaded and sealed to prevent false positive signals in the eDNA samples. Amplification negative controls consisting of HPLC water and extraction negative controls were also added in the same well location on each plate test for contamination in eDNA samples.

To confirm mitten crab presence in field samples, a subset of four positive amplifications were cloned and sequenced. Out of 21 successfully transformed clones (seven per sample), between five and seven sequences matched 100% with mitten crab on BLAST^69^, non-specific amplification was observed in remaining clones. In addition, all positive control clones (seven) for mitten crab matched 100% on BLAST.

### Statistical analysis

We employed a generalized linear modelling approach in R v.3.4.3^72^ to model detection success (i.e. the proportion of samples that tested positive for signal crayfish and mitten crabs at each site) for both water and sediment eDNA samples as a function of the number of river barriers both upstream and downstream of each positive site and river identity (n: 2 rivers; Medway and Dee). This approach aimed to test whether barriers limit the upstream or downstream migration of each species, by assessing whether an increasing number of barriers makes it more difficult to detect the species eDNA upstream or downstream. River identity also served as fragmentation status (Medway: highly fragmented, Dee: partially fragmented). We considered that either species was present at a site if two of the three PCR replicates per sample (6 samples per site) tested positive for target species. A quasibinomial log-link was used to correct for overdispersion.

## Results

### Mitten crab detection limits

Results from a 10-fold dilution series revealed that for mitten crab the limit of detection (LOD) was 0.005 ng/µl for the qPCR assay, which is the same LOD as the predetermined value for both signal crayfish and white-clawed crayfish^39^. No overlap in qPCR product tm was observed between the two species (Fig. S1; Table S3) and it was possible to detect presence of either species in a single reaction based on the diagnostic melt curve shape produced when combining varying ratios of pooled DNA for both species. Results from mixed proportions of signal crayfish and mitten crab displayed only signal crayfish melt curves from 9:1 to 7:3 µL signal crayfish: mitten crab ratios whereas from 6:4 to 1:9 µL ratios, the melt curves were diagnostic for just mitten crabs (Fig. S1; Table S3).

### Detection success and spatial distribution

Signal crayfish DNA was successfully detected in six out of the 11 sites sampled in the River Medway, whereas mitten crab DNA was only detected in three sites (Table 3; Figs S2 and S3; Tables S4 and S5). For all three catchments, both signal crayfish and mitten crab were detected within sections of the catchment where there has been visual confirmation of both species^20,65^, which confirms the utility of the essay in the field. As expected, positive sites for signal crayfish were located in the upper reaches of the river area sampled. Signal crayfish DNA was detected further downstream than previously reported in the Dee and Medway catchments^65^, however it was not possible to determine whether this represents downstream dispersal or downstream transport of eDNA from an upstream source^51,53^. Similarly, mitten crab DNA was detected further upstream than previously reported from visual surveys in the Medway and the Stour (Fig. S4; Tables S6 and S7), however, in the River Dee, mitten crab DNA was not detected at the uppermost extent of their known range^20^. Three sampling sites in the River Medway overlapped for both target species (M7, M10, M11) and similarly signal crayfish and mitten crab were detected at two of the same sites in the River Dee (D7 and D8; Figs S5 and S6; Tables S8 and S9). The River Stour also had an overlap in detection of both species in site ST3. Results from amplifying positive signal crayfish and mitten crab samples with HOT FIREPol^®^ EvaGreen^®^ qPCR multiplex indicated that there was no *A*. *astaci* present in any of the three catchments.

**Table 3:** Number of water and sediment samples collected, positive sites for both water and sediment samples for each species (signal crayfish (SC) and mitten crab (MC)) and total number of positive sites for each sample type.

Catchment	Total Water Sample Sites	Total Sediment Sample Sites	No. Positive SC sites (Water)	No. Positive SC sites (Sediment)	No. Positive MC sites (Water)	No. Positive MC sites (Sediment)
Medway	11	5	4	3	3	1
Dee	11	11	1	2	1	2
Stour	3	1	1	1	1	1
Total	25	17	6	6	5	4

### Detection success in relation to barriers

In comparison to the River Dee, the seven positive sites for signal crayfish in the Medway catchment were distributed at regular intervals down the catchment, whereas positive sites in the Dee for this species were located at very start of sample area (D1) and then clustered further downstream of three weirs (D7-D9). Despite presence of six locks within close proximity to one another in the upper Medway catchment (Fig. 1; Table S1), and three weirs situated within 15 km in the River Dee (Fig. 2; Table S1), signal crayfish DNA was detected above these barriers at M1 and D1 respectively. In contrast, despite previous records of mitten crab around Teston lock at M8, there was no DNA detected in either sediment or water samples from this site. However, there were positive detections of this target species directly upstream of Allington Lock at site M10, where mitten crab was previously known to accumulate at lock gates as has been observed in other impounded catchments^73,74^. Mitten crab DNA in the River Dee was detected at three sites upstream of Chester Weir, a barrier which is known to be passable for this species, however was not detected any further upstream than site D7.

Water and sediment samples did not perform to the same extent, with water samples producing a greater proportion of positive detections (Figs S2–S6; Tables S4–S9). For signal crayfish, there was no effect of barriers upstream or downstream (deviance = 43.31, df = 19, *P* = 0.091; Table S10) or on positive detection of the species in water samples or sediment samples (deviance = 56.05, df = 13, *P* = 0.794; Table S10). There was however an effect of river identity on positive detections of signal crayfish in water samples (deviance = 43.31, df = 19, *P* < 0.05), with a significantly higher detection success in the River Medway (highly fragmented) compared to the River Dee (partially fragmented; Table S10). For mitten crab, the number of barriers upstream of sampling site had a positive effect on detection success in water samples (deviance = 10.14, df = 19, *P* < 0.05) but not sediment samples (deviance = 17.16, df = 13, *P* = 0.997; Tables S11 and S12).

## Discussion

Here, we have identified a negative effect of barrier presence on the upstream distribution of mitten crab and apparent lack of effect of barriers in the downstream presence of signal crayfish, by comparing water eDNA detection in river catchments with differing levels of fragmentation. This effect was not observed in sediment in any of both species, which could reflect the temporal differences in detection between water and sediment^47,49^.

### Species presence compared to previous records

In all three catchments, both signal crayfish and mitten crab eDNA was detected in close proximity to locations of previous records. The exception to this being lack of mitten crab DNA near the estuary in the River Stour (ST1) and positive detections of signal crayfish in the lentic system at M11. Due to the one-way flow of eDNA in lotic systems, upstream detections of target species DNA suggest that individuals have progressed upstream; this also applies to sediment samples^53,75^. Signal crayfish are known to migrate both up and downstream anything from 1 to 4 km upstream and 1.5 to 6 km downstream per year^31^, therefore the high proportion of positive sites in the Medway catchment could suggest that this species has expanded beyond its previously considered range^76^. Similarly, positive detections of signal crayfish immediately downstream of Lake Bala in the River Dee, indicates upstream range expansion in this catchment^65^. Due to the catadromous nature of mitten crabs, juvenile crabs are known to migrate up to 750 km upstream to mature^73,74^, therefore the uppermost extent of DNA detection in a river is likely to be within close proximity to the true upstream extent of species occupation^53,77–79^. Similar to signal crayfish, within the Medway and Dee we detected mitten crab further upstream than previously reported, again suggesting upstream range expansion of the species^22^.

In both the Rivers Medway and Dee, signal crayfish and mitten crab were detected in the same sampling site, both in sediment and water samples, in locations which match the recorded downstream extent of signal crayfish and upstream extent of mitten crab^20,76^; this could indicate that both species are occupying the same stretches of the Medway and Dee around these sites, which has already been observed in other catchments in the UK^80^. Overlapping zones between signal crayfish and mitten crab are expected to result in negative impacts on local biota in comparison to single-species zones due to a combination of niche partitioning and predatory overlap^80^.

### Effect of sample type on species presence

Water and aquatic sediments are known sources of eDNA, and both sample types have been directly used in a range of non-invasive surveys and monitoring techniques^44,49,79,81^. The observed higher within-sample detection rate in sediment samples correlates with results from additional studies on DNA detection from sediment^49,82–85^, and could be a result of both the ecology of both target species and increased temporal longevity of DNA in sediment, resulting in higher detectability across sample replicates^47,49,86–88^. We found that a greater number of sampling sites were positive for target species in water samples in comparison with sediment samples. This is unexpected because both target species are benthic by nature, it was expected that eDNA would be more likely to be detected in the sediments than in surface waters, as aqueous DNA from crabs and crayfish is most likely to originate from faeces which sink rapidly into the substratum^87–89^, due to lack of mucus exuded^46,73,86,88^. The conditions of the aquatic sediment can enable DNA to remain detectable for a longer period of time (at least 132 days for fish vs 25 days in water samples^49^), in comparison to DNA free in aqueous solution. Due to the varying temporal persistence of DNA in sediment^89^, it is difficult to determine the time of DNA deposition, which can be a problem for assessing current presence/absence, as depositions from past occupancy can result in false positives for target species^75,90^. There is little information regarding the longevity of invertebrate eDNA in sediments, however due to the cross-over in detection success between sediment and water samples, it is likely that detections in sediment for signal crayfish and mitten crabs represent more current-occupancy than past-occupancy^49,87^.

Here, we have validated the use of HRM for the analysis of field eDNA samples (as opposed to use for optimisation only^40,91^), which is still incipient, both for water and sediment, although this method has been extensively used as a highly discriminative method for identifying species^39,92,93^. We have observed that the use of the same primers can present problems when the amount of eDNA from one of the species is considerably larger than for the other. To overcome this problem, we used a large number of replicates per sampling location and sequenced the resulting PCR in cases when a single species was detected. When collecting a large number of replicates is not possible, optimisation of specific primers may be the best alternative approach.

### Barrier influence on presence and DNA detectability

The presence of the locks and flood gates in the River Medway appeared to have an influence over the DNA detection of mitten crab in this river. Detection likelihood of mitten crab increased with the number of barriers upstream of the sampling site, which indicates that barriers in the Medway are restricting the upstream movement of this species^34^. During their upstream migration, mitten crabs have been known to aggregate at barriers, especially when banks are too steep to navigate around barrier on land and the presence of large structures such as dams and flood gates are known to considerably slow down its upstream migration^73,74^. This congregation of individuals is likely to result in a stronger eDNA signal further downstream, because density is known to be the major contributing factor to successful DNA detection in numerous aquatic species^53,79,81,94^. The most upstream record of mitten crabs was in the River Beult, a tributary of the River Medway, which branches off from the main river ~5 km before of a series of six consecutive locks. Our detections of mitten crab DNA from water samples taken in this tributary, suggests this species is present here as opposed to the main river as no detections were found any further upstream of the River Medway. In contrast with the mitten crab, we found that the barriers did not affect the presence of the signal crayfish, probably due to the fact signal crayfish mainly disperse in a downstream direction^26,66^. In the River Stour, mitten crab appears to have expanded its range as this species was detected alongside signal crayfish in the most upstream site sampled. Mitten crabs had previously only been reported in the mouth of the estuary in this river system, and successful upstream range expansion could be as a result of the high levels of connectivity in the Stour.

Significantly higher detection success for signal crayfish in the River Medway compared to the River Dee could be the result of varying hydrological conditions, crayfish abundance or the difference in seasonality between the sampling period for each river^43,44,48,68^. Some studies have reported reduction of DNA detection for signal crayfish in the winter months (November – February^43^) due to the winter torpor signal crayfish undergo as part of their annual life cycle^43,87,95^. Temperature is considered to be the main driver for reduction in crayfish activity^95^, which can directly correspond to the amount of eDNA being released into the local environment^43,85^. However, our previous work on signal crayfish eDNA during October resulted in the species being detected in all reported locations, suggesting a substantial level of detection during the autumn^39^. Additionally, temperatures in the River Medway and River Dee were not very different between July (average 16.1 °C across all sampling sites) and October (average 14.1 °C across all sampling sites; Table 1), and therefore we expected the levels of crayfish activity, and eDNA shedding rates, to be comparable^87^. It is thus unlikely that the difference in detection rate of signal crayfish DNA is as a direct result of the seasonality in this case.

The detectability of eDNA in a flowing river depends on both biotic and abiotic factors such as distance from source^53,56^, water velocity^53,55,56,94^, and temperature^51,53,94,96^. The presence of a series of locks along a section of river, as seen in the River Medway, could have the potential to create ‘mini-lentic systems’ upstream of each obstacle^94^, and therefore eDNA is more likely to settle and bind to sediment^49,55^, as opposed to being carried downstream^55^. Further research into the fate of DNA in fragmented river systems should be investigated to address this concept.

Overall, assessing the influence of barriers on invasive species presence and distribution is important for informing management strategies^97–99^. Long-term persistence of mitten crabs depends on the ability of juveniles to migrate upstream and colonise suitable freshwater habitats^22,77,100^, therefore river obstacles can have a great influence over colonisation success^34^. Additionally, being able to detect sites of predicted range overlap between signal crayfish and mitten crabs using eDNA is important for informing management strategies of critical areas for invasive species control, particularly for species which experience complex trophic interactions and are potentially synergistic^23,33,80,101,102^. Our work suggests that sampling in the proximity of obstacles can increase species detectability through eDNA, particularly for species like the mitten crab which tend to concentrate downstream of the barriers. We also found that water samples can outperform sediment samples for DNA detection of benthic species, highlighting the ability to detect sufficient quantities of DNA in flowing systems to determine current distribution.

## Supplementary information

Supplementary information

## Data Availability

All data generated or analysed during this study are included in this published article (and its Supplementary Information files).
