# Supplementary material for: Effect of artificial barriers on the distribution of the invasive signal crayfish and Chinese mitten crab
Source: Sci Rep. 2019 May 10;9:7230. doi: 10.1038/s41598-019-43570-3 (PMC6510734; doi:10.1038/s41598-019-43570-3)
Supplement: Supplementary file 1 — Supplementary information [file 41598_2019_43570_MOESM1_ESM.docx]

Effect of artificial barriers on the distribution of the invasive signal crayfish and Chinese mitten crab

Chloe Victoria Robinson^1^, Carlos Garcia de Leaniz^1^, Sofia Consuegra^1^.

^1^ Bioscience Department, Swansea University, Singleton Park, Swansea, United Kingdom.

Corresponding Author:

Sofia Consuegra^1^

Email address: [s.consuegra@swansea.ac.uk](mailto:s.consuegra@swansea.ac.uk)

**Supplementary Information**

**Table S1.** Information on location of known major barriers present within the upstream to downstream sampling area of the River Medway, River Dee and River Stour, including type of and GPS of obstruction, distance to nearest upstream and downstream barriers (km) based on information provided by Environment Agency and Natural Resources Wales (EA, 2017).

| River | Obstruction Name | Obstruction Type | Obstruction GPS | Distance upstream to next barrier (km) | Distance downstream to next barrier (km) |
| --- | --- | --- | --- | --- | --- |
| Medway | Leigh Barrier Control Structure | Flood Gate | TQ 56386 46116 | N/A | 3.5 |
| Medway | Town Lock | Lock | TQ 59356 46478 | 3.5 | 2.2 |
| Medway | Eldridges Lock | Lock | TQ 61191 47173 | 2.2 | 1.4 |
| Medway | Porters Lock | Lock | TQ 62488 47088 | 1.4 | 1.9 |
| Medway | East Lock | Lock | TQ 64202 47298 | 1.9 | 1.2 |
| Medway | Oak Weir Lock | Lock | TQ 65354 47202 | 1.2 | 2.0 |
| Medway | Sluice Weir Lock | Lock | TQ 66919 47947 | 2.0 | 4.6 |
| Medway | Hampstead Lock | Lock | TQ 68686 50345 | 4.6 | 4.7 |
| Medway | Teston Lock | Lock | TQ 70840 53052 | 4.7 | 3.2 |
| Medway | East Farleigh Lock | Lock | TQ 73521 53572 | 3.2 | 3.5 |
| Medway | Allington Lock | Lock | TQ 74833 58153 | 3.7 | 2.3 |
| Medway | Tidal gate 2 | Flood Gate | TQ 72961 58962 | 2.3 | 0.2 |
| Medway | Tidal gate 4 | Flood Gate | TQ 72751 58847 | 0.2 | 1.0 |
| Medway | Tidal gate 6 | Flood Gate | TQ 71750 58994 | 1.0 | N/A |
| Dee | Horseshoe falls | Weir | SJ 19540 43330 | N/A | 23.8 |
| Dee | Weir on the Dee | Weir | SJ 34841 41469 | 23.8 | 4.0 |
| Dee | Erbistock Mill Weir | Weir | SJ 35423 42107 | 4.0 | 53.1 |
| Dee | Chester Weir | Weir | SJ 40757 65819 | 53.1 | N/A |

**Table S2.** Upstream and downstream proximity of each sampling in relation to the nearest known river obstruction in the River Medway and River Dee.

| River | Site | Distance upstream to next barrier (km) | Distance downstream to next barrier (km) |
| --- | --- | --- | --- |
| Medway | M1 | 3.3 | 0.3 |
| Medway | M2 | 2.0 | 4.0 |
| Medway | M3 | N/A | 3.9 |
| Medway | M4 | 3.4 | 0.6 |
| Medway | M5 | N/A | 5.9 |
| Medway | M6 | N/A | 13.6 |
| Medway | M7 | N/A | 8.6 |
| Medway | M8 | 0.2 | 3.0 |
| Medway | M9 | 3.3 | 0.04 |
| Medway | M10 | 3.3 | 0.2 |
| Medway | M11 | 7.5 | 5.0 |
| Dee | D1 | N/A | 42.7 |
| Dee | D2 | N/A | 30.3 |
| Dee | D3 | N/A | 30.2 |
| Dee | D4 | 1.1 | 26.8 |
| Dee | D5 | 16.4 | 7.3 |
| Dee | D6 | 2.5 | 49.2 |
| Dee | D7 | 16.6 | 32.6 |
| Dee | D8 | 24.1 | 20.3 |
| Dee | D9 | 36.6 | 11.6 |
| Dee | D10 | 44.2 | 5.4 |
| Dee | D11 | 48.7 | 1.1 |

**Table S3.** Melt peak data from SsoFast™ EvaGreen® dilution series for *Pacifastacus leniusculus* (SC), and *Eriocheir sinensis* (MC) mixed proportions.

| Sample | Fluorophore | Melt Temperature (^o^C) |
| --- | --- | --- |
| 9:1 | SYBR | 73.7 |
| 9:1 | SYBR | 73.7 |
| 9:1 | SYBR | 73.7 |
| 8:2 | SYBR | 73.6 |
| 8:2 | SYBR | 736. |
| 8:2 | SYBR | 73.6 |
| 7:3 | SYBR | 73.6 |
| 7:3 | SYBR | 73.6 |
| 7:3 | SYBR | 73.6 |
| 6:4 | SYBR | 73.4 |
| 6:4 | SYBR | 73.4 |
| 6:4 | SYBR | 73.4 |
| 5:5 | SYBR | 73.4 |
| 5:5 | SYBR | 73.4 |
| 5:5 | SYBR | 73.4 |
| 4:6 | SYBR | 73.4 |
| 4:6 | SYBR | 73.4 |
| 4:6 | SYBR | 73.4 |
| 3:7 | SYBR | 73.4 |
| 3:7 | SYBR | 73.4 |
| 3:7 | SYBR | 73.4 |
| 2:8 | SYBR | 73.4 |
| 2:8 | SYBR | 73.4 |
| 2:8 | SYBR | 73.4 |
| 1:9 | SYBR | 73.4 |
| 1:9 | SYBR | 73.4 |
| 1:9 | SYBR | 73.4 |
| PC_SC | SYBR | 73.7 |
| PC_SC | SYBR | 73.7 |
| PC_SC | SYBR | 73.7 |
| PC_MC | SYBR | 73.3 |
| PC_MC | SYBR | 73.4 |
| PC_MC | SYBR | 73.3 |
| MB | SYBR | None |
| MB | SYBR | None |
| MB | SYBR | None |

.

Sample ID: PC_SC Signal crayfish positive DNA control, PC_MC Mitten crab

T**able S4**. Melt peak data from SsoFast™ EvaGreen® water field samples in the River Medway positive for *Pacifastacus leniusculus* (SC), and *Eriocheir sinensis* (MC) DNA.

| Fluorophore | Sample | Melt Temperature (^o^C) |
| --- | --- | --- |
| SYBR | M_1A | 73.9 |
| SYBR | M_1A | 73.8 |
| SYBR | M_1A | 73.7 |
| SYBR | M_1C | 73.8 |
| SYBR | M_1C | 73.7 |
| SYBR | M_1C | 73.7 |
| SYBR | M_1D | 73.7 |
| SYBR | M_1D | 73.6 |
| SYBR | M_1D | 73.6 |
| SYBR | M_1F | 73.7 |
| SYBR | M_1F | 73.6 |
| SYBR | M_1F | 73.6 |
| SYBR | M_5A | 74.0 |
| SYBR | M_5A | 74.1 |
| SYBR | M_5A | 74.1 |
| SYBR | M_5B | 74.0 |
| SYBR | M_5B | 74.0 |
| SYBR | M_5C | 74.1 |
| SYBR | M_5C | 74.1 |
| SYBR | M_5D | 74.1 |
| SYBR | M_5D | 74.0 |
| SYBR | M_5D | 74.1 |
| SYBR | M_5E | 74.0 |
| SYBR | M_5E | 73.8 |
| SYBR | M_5E | 73.8 |
| SYBR | M_5F | 73.8 |
| SYBR | M_5F | 73.8 |
| SYBR | M_5F | 74.1 |
| SYBR | M_7A | 73.9 |
| SYBR | M_7A | 72.9 |
| SYBR | M_7E | 73.6 |
| SYBR | M_7E | 73.6 |
| SYBR | M_7F | 73.3 |
| SYBR | M_7F | 73.4 |
| SYBR | M_7F | 73.3 |
| SYBR | M_10A | 73.9 |
| SYBR | M_10A | 73.6 |
| SYBR | M_10A | 73.7 |
| SYBR | M_10C | 73.3 |
| SYBR | M_10C | 73.3 |
| SYBR | M_10D | 73.6 |
| SYBR | M_10D | 73.7 |
| SYBR | M_11A | 73.5 |
| SYBR | M_11A | 73.5 |
| SYBR | M_11B | 73.4 |
| SYBR | M_11B | 73.4 |
| SYBR | M_1BL | None |
| SYBR | M_1BL | None |
| SYBR | M_1BL | None |
| SYBR | M_2BL | None |
| SYBR | M_2BL | None |
| SYBR | M_2BL | None |
| SYBR | M_3BL | None |
| SYBR | M_3BL | None |
| SYBR | M_3BL | None |
| SYBR | M_4BL | None |
| SYBR | M_4BL | None |
| SYBR | M_4BL | None |
| SYBR | M_5BL | None |
| SYBR | M_5BL | None |
| SYBR | M_5BL | None |
| SYBR | M_6BL | None |
| SYBR | M_6BL | None |
| SYBR | M_6BL | None |
| SYBR | M_7BL | None |
| SYBR | M_7BL | None |
| SYBR | M_7BL | None |
| SYBR | M_8BL | None |
| SYBR | M_8BL | None |
| SYBR | M_8BL | None |
| SYBR | M_9BL | None |
| SYBR | M_9BL | None |
| SYBR | M_9BL | None |
| SYBR | M_10BL | None |
| SYBR | M_10BL | None |
| SYBR | M_10BL | None |
| SYBR | M_11BL | None |
| SYBR | M_11BL | None |
| SYBR | M_11BL | None |
| SYBR | EB1 | None |
| SYBR | EB1 | None |
| SYBR | EB1 | None |
| SYBR | EB2 | None |
| SYBR | EB2 | None |
| SYBR | EB2 | None |
| SYBR | EB3 | None |
| SYBR | EB3 | None |
| SYBR | EB3 | None |
| SYBR | MB | None |
| SYBR | MB | None |
| SYBR | MB | None |
| SYBR | PC_MC | 73.3 |
| SYBR | PC_MC | 73.3 |
| SYBR | PC_SC | 73.7 |
| SYBR | PC_SC | 73.7 |

Sample ID: M_# Medway catchment sample with corresponding subsample letter, PC_SC Signal crayfish positive DNA control, PC_MC Mitten crab positive DNA control, EB# extraction blank, M_#BL site blank, MB amplification blank

**Table S5**. Melt peak data from SsoFast™ EvaGreen® sediment field samples in the River Medway positive for *Pacifastacus leniusculus* (SC), and *Eriocheir sinensis* (MC) DNA.

| Fluorophore | Sample | Melt Temperature (^o^C) |
| --- | --- | --- |
| SYBR | M_S7_B | 73.7 |
| SYBR | M_S7_B | 73.7 |
| SYBR | M_S7_B | 73.7 |
| SYBR | M_S7_C | 73.8 |
| SYBR | M_S7_C | 74.0 |
| SYBR | M_S7_C | 73.8 |
| SYBR | M_S7_E | 73.9 |
| SYBR | M_S7_E | 73.8 |
| SYBR | M_S7_E | 73.6 |
| SYBR | M_S7_F | 73.6 |
| SYBR | M_S7_F | 73.6 |
| SYBR | M_S7_F | 73.9 |
| SYBR | M_S8_A | 73.9 |
| SYBR | M_S8_A | 74.0 |
| SYBR | M_S8_A | 74.0 |
| SYBR | M_S8_C | 73.6 |
| SYBR | M_S8_C | 73.8 |
| SYBR | M_S8_D | 73.9 |
| SYBR | M_S8_D | 73.9 |
| SYBR | M_S8_D | 73.9 |
| SYBR | M_S8_F | 73.6 |
| SYBR | M_S8_F | 73.7 |
| SYBR | M_S11_A | 73.3 |
| SYBR | M_S11_A | 73.3 |
| SYBR | M_S11_A | 73.3 |
| SYBR | M_S11_B | 73.3 |
| SYBR | M_S11_B | 73.4 |
| SYBR | M_S11_B | 73.4 |
| SYBR | M_S11_C | 73.1 |
| SYBR | M_S11_C | 73.2 |
| SYBR | M_S11_C | 75.3 |
| SYBR | M_S11_E | 73.6 |
| SYBR | M_S11_E | 73.7 |
| SYBR | M_S1BL | None |
| SYBR | M_S1BL | None |
| SYBR | M_S1BL | None |
| SYBR | M_S2BL | None |
| SYBR | M_S2BL | None |
| SYBR | M_S2BL | None |
| SYBR | M_S3BL | None |
| SYBR | M_S3BL | None |
| SYBR | M_S3BL | None |
| SYBR | M_S4BL | None |
| SYBR | M_S4BL | None |
| SYBR | M_S4BL | None |
| SYBR | M_S5BL | None |
| SYBR | M_S5BL | None |
| SYBR | M_S5BL | None |
| SYBR | PC_SC | 73.9 |
| SYBR | PC_SC | 73.9 |
| SYBR | PC_SC | 74 |
| SYBR | PC_MC | 73.3 |
| SYBR | PC_MC | 73.4 |
| SYBR | PC_MC | 73.3 |
| SYBR | MB | None |
| SYBR | MB | None |
| SYBR | MB | None |

Sample ID: M_S# Medway catchment sample with corresponding subsample letter, PC_SC Signal crayfish positive DNA control, PC_MC Mitten crab positive DNA control, EB extraction blank, M_S#BL site blank, MB amplification blank

**Table S6**. Melt peak data from SsoFast™ EvaGreen® water field samples in the River Stour positive for *Pacifastacus leniusculus* (SC), and *Eriocheir sinensis* (MC) DNA.

| Fluorophore | Sample | Melt Temperature (^o^C) |
| --- | --- | --- |
| SYBR | S_3A | 73.8 |
| SYBR | S_3A | 73.6 |
| SYBR | S_3A | 73.8 |
| SYBR | S_3B | 73.4 |
| SYBR | S_3B | 73.4 |
| SYBR | S_3B | 73.3 |
| SYBR | S_1BL | None |
| SYBR | S_1BL | None |
| SYBR | S_1BL | None |
| SYBR | S_2BL | None |
| SYBR | S_2BL | None |
| SYBR | S_2BL | None |
| SYBR | S_3BL | None |
| SYBR | S_3BL | None |
| SYBR | S_3BL | None |
| SYBR | PC_MC | 73.4 |
| SYBR | PC_MC | 73.3 |
| SYBR | PC_MC | 73.3 |
| SYBR | PC_SC | 73.7 |
| SYBR | PC_SC | 73.7 |
| SYBR | PC_SC | 74.0 |
| SYBR | MB | None |
| SYBR | MB | None |
| SYBR | MB | None |

Sample ID: S_# Stour catchment sample with corresponding subsample letter, PC_SC Signal crayfish positive DNA control, PC_MC Mitten crab positive DNA control, EB extraction blank, MB amplification blank

**Table S7**. Melt peak data from SsoFast™ EvaGreen® sediment field samples in the River Stour positive for *Pacifastacus leniusculus* (SC), and *Eriocheir sinensis* (MC) DNA.

| Fluorophore | Sample | Melt Temperature (^o^C) |
| --- | --- | --- |
| SYBR | S_S_3B | 73.6 |
| SYBR | S_S_3B | 73.6 |
| SYBR | S_S_3D | 73.7 |
| SYBR | S_S_3D | 73.7 |
| SYBR | S_S_3E | 73.6 |
| SYBR | S_S_3E | 73.7 |
| SYBR | S_S_3E | 74.0 |
| SYBR | S_S_3F | 73.6 |
| SYBR | S_S_3F | 73.6 |
| SYBR | MB | None |
| SYBR | MB | None |
| SYBR | MB | None |
| SYBR | PC_MC | 73.4 |
| SYBR | PC_MC | 73.3 |
| SYBR | PC_MC | 73.3 |
| SYBR | PC_SC | 73.9 |
| SYBR | PC_SC | 74.0 |
| SYBR | PC_SC | 74.0 |

Sample ID: S_S_# Stour catchment sample with corresponding subsample letter, PC_SC Signal crayfish positive DNA control, PC_MC Mitten crab positive DNA control, EB extraction blank, MB amplification blank

**Table S8**. Melt peak data from SsoFast™ EvaGreen® water field samples in the River Dee positive for *Pacifastacus leniusculus* (SC), and *Eriocheir sinensis* (MC) DNA.

| Fluorophore | Sample | Melt Temperature (^o^C) |
| --- | --- | --- |
| SYBR | D_1D | 73.9 |
| SYBR | D_1D | 73.6 |
| SYBR | D_7E | 73.9 |
| SYBR | D_7E | 73.8 |
| SYBR | D_9D | 73.5 |
| SYBR | D_9D | 73.4 |
| SYBR | D_9D | 73.3 |
| SYBR | D_1BL | None |
| SYBR | D_1BL | None |
| SYBR | D_1BL | None |
| SYBR | D_2BL | None |
| SYBR | D_2BL | None |
| SYBR | D_2BL | None |
| SYBR | D_3BL | None |
| SYBR | D_3BL | None |
| SYBR | D_3BL | None |
| SYBR | D_4BL | None |
| SYBR | D_4BL | None |
| SYBR | D_4BL | None |
| SYBR | D_5BL | None |
| SYBR | D_5BL | None |
| SYBR | D_5BL | None |
| SYBR | D_6BL | None |
| SYBR | D_6BL | None |
| SYBR | D_6BL | None |
| SYBR | D_7BL | None |
| SYBR | D_7BL | None |
| SYBR | D_7BL | None |
| SYBR | D_8BL | None |
| SYBR | D_8BL | None |
| SYBR | D_8BL | None |
| SYBR | D_9BL | None |
| SYBR | D_9BL | None |
| SYBR | D_9BL | None |
| SYBR | D_10BL | None |
| SYBR | D_10BL | None |
| SYBR | D_10BL | None |
| SYBR | D_11BL | None |
| SYBR | D_11BL | None |
| SYBR | D_11BL | None |
| SYBR | EB4 | None |
| SYBR | EB4 | None |
| SYBR | EB4 | None |
| SYBR | EB5 | None |
| SYBR | EB5 | None |
| SYBR | EB5 | None |
| SYBR | EB6 | None |
| SYBR | EB6 | None |
| SYBR | EB6 | None |
| SYBR | MB | None |
| SYBR | MB | None |
| SYBR | MB | None |
| SYBR | PC_MC | 73.5 |
| SYBR | PC_MC | 73.5 |
| SYBR | PC_MC | 73.5 |
| SYBR | PC_SC | 73.7 |
| SYBR | PC_SC | 73.7 |
| SYBR | PC_SC | 74.0 |

Sample ID: D_# Dee catchment sample with corresponding subsample letter, PC_SC Signal crayfish positive DNA control, PC_MC Mitten crab positive DNA control, EB extraction blank, D_#BL Dee site blank, EB# extraction blank, MB amplification blank

**Table S9**. Melt peak data from SsoFast™ EvaGreen® sediment field samples in the River Dee positive for *Pacifastacus leniusculus* (SC), and *Eriocheir sinensis* (MC) DNA.

| Fluorophore | Sample | Melt Temperature (^o^C) |
| --- | --- | --- |
| SYBR | D1_S_A | 73.9 |
| SYBR | D1_S_A | 73.9 |
| SYBR | D1_S_A | 73.9 |
| SYBR | D1_S_B | 73.9 |
| SYBR | D1_S_B | 73.8 |
| SYBR | D1_S_B | 73.9 |
| SYBR | D1_S_C | 73.7 |
| SYBR | D1_S_C | 73.7 |
| SYBR | D1_S_D | 73.7 |
| SYBR | D1_S_D | 73.9 |
| SYBR | D1_S_E | 74.0 |
| SYBR | D1_S_E | 73.7 |
| SYBR | D1_S_F | 74.0 |
| SYBR | D1_S_F | 73.6 |
| SYBR | D7_S_A | 73.2 |
| SYBR | D7_S_A | 73.2 |
| SYBR | D7_S_A | 73.3 |
| SYBR | D7_S_C | 73.4 |
| SYBR | D7_S_C | 73.4 |
| SYBR | D8_S_A | 73.2 |
| SYBR | D8_S_A | 73.4 |
| SYBR | D8_S_B | 73.2 |
| SYBR | D8_S_B | 73.2 |
| SYBR | D8_S_B | 73.2 |
| SYBR | D8_S_C | 73.8 |
| SYBR | D8_S_C | 73.9 |
| SYBR | D8_S_D | 73.6 |
| SYBR | D8_S_D | 73.6 |
| SYBR | D8_S_E | 73.3 |
| SYBR | D8_S_E | 73.4 |
| SYBR | D8_S_F | 73.7 |
| SYBR | D8_S_F | 73.7 |
| SYBR | D_BL1 | None |
| SYBR | D_BL1 | None |
| SYBR | D_BL1 | None |
| SYBR | D_BL2 | None |
| SYBR | D_BL2 | None |
| SYBR | D_BL2 | None |
| SYBR | D_BL3 | None |
| SYBR | D_BL3 | None |
| SYBR | D_BL3 | None |
| SYBR | D_BL4 | None |
| SYBR | D_BL4 | None |
| SYBR | D_BL4 | None |
| SYBR | MB | None |
| SYBR | MB | None |
| SYBR | MB | None |
| SYBR | PC_MC | 73.4 |
| SYBR | PC_MC | 73.4 |
| SYBR | PC_MC | 73.5 |
| SYBR | PC_SC | 73.7 |
| SYBR | PC_SC | 73.8 |
| SYBR | PC_SC | 73.8 |

Sample ID: D#_S Dee catchment sample with corresponding subsample letter, PC_SC Signal crayfish positive DNA control, PC_MC Mitten crab positive DNA control, EB extraction blank, D_BL# Dee site blank, EB# extraction blank, MB amplification blank

**Table S10**. Statistical output for signal crayfish (*Pacifastacus leniusculus*) binomial and quasibinomial refit models, using proportion of positive eDNA samples for both water and sediment samples.

| Model | Factor | Formula | Coefficients | z value | p-value | df | Residual deviance | Dispersion parameter | AIC |
| --- | --- | --- | --- | --- | --- | --- | --- | --- | --- |
| Binomial | No. barriers upstream | WaterSC~River+No.barriersUpstream | River | 3.872 | 0.0001 | 19 | 43.314 | 1.00 | 59.627 |
|  |  |  | No.barriersUpstream | -2.722 | 0.006 |  |  |  |  |
| Quasibinomial |  | WaterSC~River+No.barriersUpstream | River | 2.533 | 0.020 |  |  | 2.34 | NA |
|  |  |  | No.barriersUpstream | -1.781 | 0.091 |  |  |  |  |
|  |  |  |  |  |  |  |  |  |  |
| Binomial | No. barriers downstream | WaterSC~River+No.barriersDownstream | River | 0.776 | 0.438 | 19 | 43.314 | 1.00 | 59.627 |
|  |  |  | No.barriersDownstream | 2.722 | 0.007 |  |  |  |  |
| Quasibinomial |  | WaterSC~River+No.barriersDownstream | River | 0.508 | 0.617 | 19 |  | 2.34 | NA |
|  |  |  | No.barriersDownstream | 1.781 | 0.091 |  |  |  |  |
|  |  |  |  |  |  |  |  |  |  |
| Binomial | No. barriers upstream | SedimentSC~River+No.barriersUpstream | River | 1.711 | 0.087 | 13 | 56.046 | 1.00 | 70.935 |
|  |  |  | No.barriersUpstream | -0.577 | 0.564 |  |  |  |  |
| Quasibinomial |  | SedimentSC~River+No.barriersUpstream | River | 0.790 | 0.075 |  |  | 4.69 | NA |
|  |  |  | No.barriersUpstream | -0.266 | 0.794 |  |  |  |  |
|  |  |  |  |  |  |  |  |  |  |
| Binomial | No. barriers downstream | SedimentSC~River+No.barriersDownsstream | River | 1.541 | 0.123 | 13 | 56.046 | 1.00 | 70.935 |
|  |  |  | No.barriersDownstream | 0.577 | 0.564 |  |  |  |  |
| Quasibinomial |  | SedimentSC~River+No.barriersDownsstream | River | 0.712 | 0.489 |  |  | 4.69 | NA |
|  |  |  | No.barriersDownstream | 0.266 | 0.794 |  |  |  |  |

| Model | Factor | Formula | Coefficients | z value | p-value | df | Residual deviance | Dispersion parameter | AIC |
| --- | --- | --- | --- | --- | --- | --- | --- | --- | --- |
| Binomial | No. barriers Upstream | WaterMC~River+No.barriersUpstream | River | -0.955 | 0.339 | 19 | 10.136 | 1.00 | 23.827 |
|  |  |  | No.barriersUpstream | 2.392 | 0.017 |  |  |  |  |
| Quasibinomial |  | WaterMC~River+No.barriersUpstream | River | -1.067 | 0.299 |  |  | 0.80 | NA |
|  |  |  | No.barriersUpstream | 2.674 | 0.015 |  |  |  |  |
|  |  |  |  |  |  |  |  |  |  |
| Binomial | No. barriers downstream | WaterMC~River+No.barriersDownstream | River | 2.240 | 0.025 | 19 | 10.136 | 1.00 | 23.827 |
|  |  |  | No.barriersDownstream | -2.392 | 0.017 |  |  |  |  |
| Quasibinomial |  | WaterMC~River+No.barriersDownstream | River | 2.504 | 0.022 |  |  | 0.800 | NA |
|  |  |  | No.barriersDownstream | -2.674 | 0.015 |  |  |  |  |
|  |  |  |  |  |  |  |  |  |  |
| Binomial | No. barriers upstream | WaterMC~River+No.barriersUpstream | River | -0.005 | 0.996 | 13 | 17.164 | 1.00 | 29.83 |
|  |  |  | No.barriersUpstream | 0.005 | 0.996 |  |  |  |  |
| Quasibinomial |  | WaterMC~River+No.barriersUpstream | River | -0.004 | 0.997 |  |  | 1.292 | NA |
|  |  |  | No.barriersUpstream | 0.004 | 0.997 |  |  |  |  |
|  |  |  |  |  |  |  |  |  |  |
| Binomial | No. barriers downstream | SedimentMC~River+No.barriersDownstream | River | -0.005 | 0.996 | 13 | 17.164 | 1.00 | 29.83 |
|  |  |  | No.barrriersDownstream | -0.005 | 0.996 |  |  |  |  |
| Quasibinomial |  | SedimentMC~River+No.barriersDownstream | River | -0.004 | 0.997 |  |  | 1.292 | NA |
|  |  |  | No.barriersDownstream | -0.004 | 0.997 |  |  |  |  |

**Table S11**. Statistical output for Chinese mitten crab (*Eriocheir sinensis*) binomial and quasibinomial refit models, using proportion of positive eDNA samples for both water and sediment samples.

**Table S12**. Statistical output for Chinese mitten crab (*Eriocheir sinensis*) Spearman’s rank correlation for proportion of positive water eDNA samples with number of barriers upstream.

| Model | Formula | S | p-value | rho estimates |
| --- | --- | --- | --- | --- |
| Spearman’s rank correlation rho | No.barriersUpstream and WaterMC/(Water.pos.MC+Water.neg.MC) | 1018.9 | 0.048 | 0.425 |
| Spearman’s rank correlation rho | No.barriersDownstream and WaterMC/(Water.posMC+Water.neg.MC) | 2156.4 | 0.331 | -0.218 |


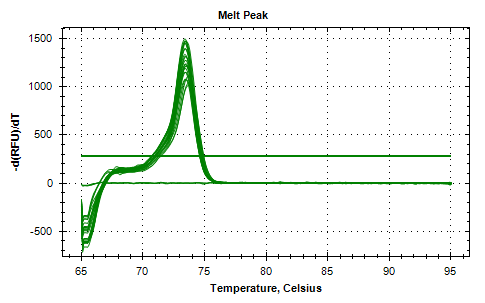
**Figure S1.** Melt peaks from SsoFast™ EvaGreen® mixed proportions for *Pacifastacus leniusculus*, and *Eriocheir sinensis* DNA ratios 9:1 to 1:9 µL signal crayfish: mitten crab DNA and positive control pools for each species.


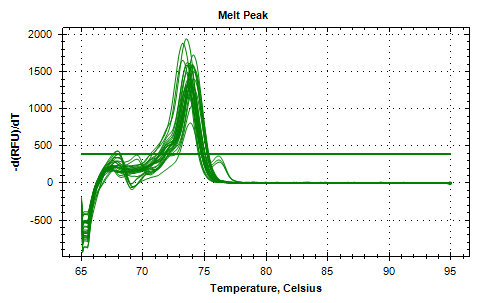

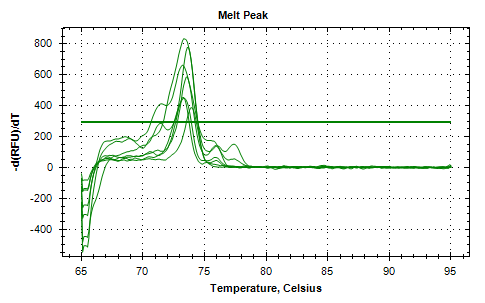

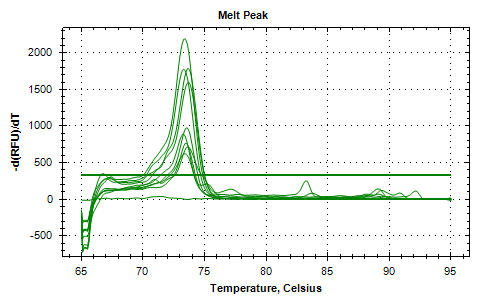


**Figure S2**. Melt peaks from SsoFast™ EvaGreen® water field samples in the River Medway positive for *Pacifastacus leniusculus*, and *Eriocheir sinensis* DNA.


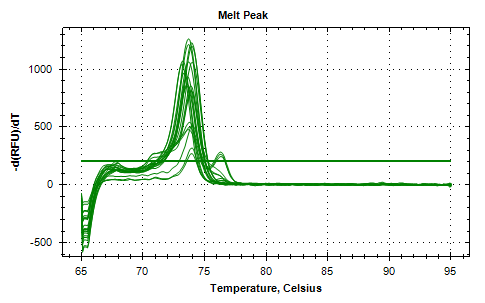

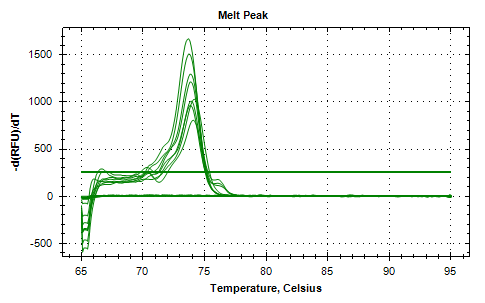


**Figure S3.** Melt peaks from SsoFast™ EvaGreen® sediment field samples in the River Medway positive for *Pacifastacus leniusculus*, and *Eriocheir sinensis* DNA.


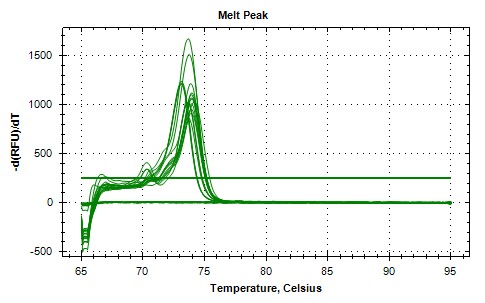

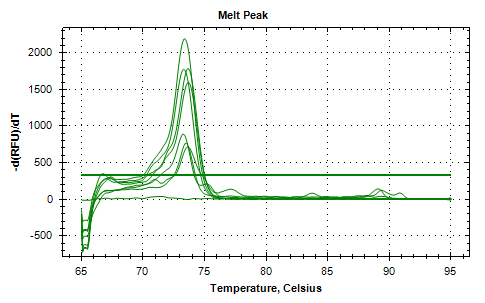


**A**

**B**

**Figure S4**. Melt peaks from SsoFast™ EvaGreen® water (A) and sediment (B) field samples in the River Stour positive for *Pacifastacus leniusculus*, and *Eriocheir sinensis* DNA.


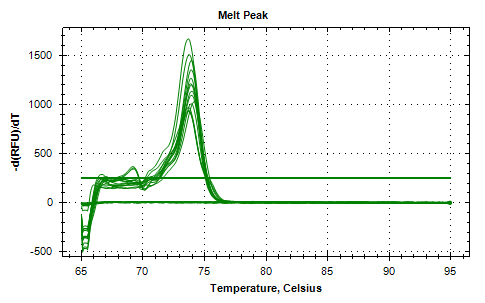

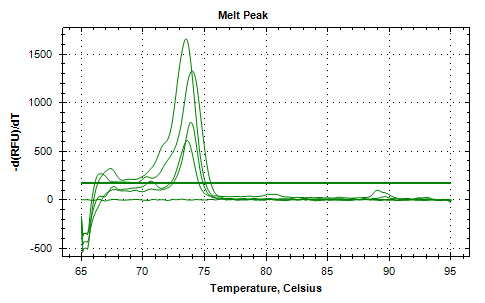

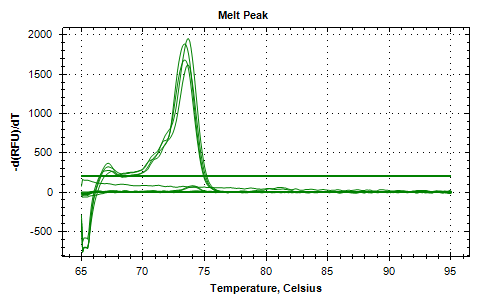


**Figure S5**. Melt peaks from SsoFast™ EvaGreen® water field samples in the River Dee positive for *Pacifastacus leniusculus*, and *Eriocheir sinensis* DNA.


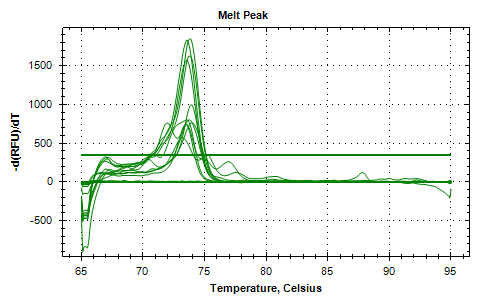

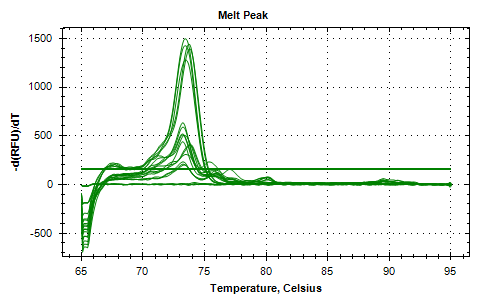

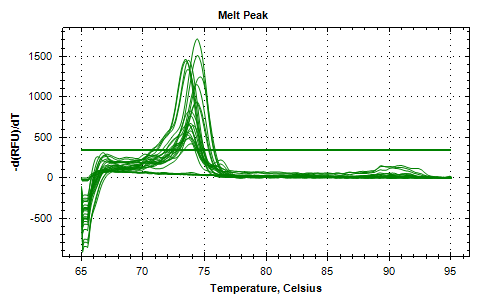


**Figure S6**. Melt peaks from SsoFast™ EvaGreen® sediment field samples in the River Dee positive for *Pacifastacus leniusculus*, and *Eriocheir sinensis* DNA.
